# Age-related differences in men’s preferences and barriers to healthcare: Insights from a national Australian survey

**DOI:** 10.1371/journal.pone.0323733

**Published:** 2025-05-23

**Authors:** Robert Palmer, James Kite, Philayrath Phongsavan, Timothy J. Moss, Bernie Marshall, Nicole Halim, Ben J. Smith

**Affiliations:** 1 Prevention Research Collaboration, Sydney School of Public Health, Faculty of Medicine and Health, The University of Sydney, Sydney, NSW, Australia; 2 Charles Perkins Centre, The University of Sydney, Sydney, NSW, Australia; 3 Healthy Male, Level 2, Melbourne, Victoria, Australia; 4 Department of Obstetrics and Gynaecology, Monash University, Clayton, Victoria, Australia; 5 School of Health and Social Development, Deakin University, Burwood, Victoria, Australia; University of Sharjah College of Health Sciences, UNITED ARAB EMIRATES

## Abstract

**Objectives:**

The high burden of preventable disease among men in many countries has highlighted the urgency of promoting stronger engagement by men in health services and programs. In order to inform prevention and early intervention strategies in Australia, this study aimed to examine how age and other socio-demographic factors moderate help-seeking preferences among men in this population, and the major psychosocial and practical barriers to healthcare use for men across the life course.

**Design:**

Cross-sectional survey using a nationally representative sample.

**Setting:**

Online survey in March 2021.

**Participants:**

English-speaking Australian men aged 18-years and older, recruited using a probabilistic sampling method. Of the 1,409 men invited to participate, 1,282 (91%) completed the survey.

**Main outcome measures:**

Preferences for help-seeking related to physical and mental health, and psychosocial and practical barriers to help-seeking.

**Results:**

Compared to men aged 70 years and over, younger men were less likely to choose professional sources of help, with those aged 18–29 years showing the lowest odds when needing assistance for physical (OR = 0.28, 95% CI 0.17–0.49) and mental health (OR = 0.28, 95% CI 0.16–0.46). Men in this youngest age group also reported experiencing more practical barriers to healthcare access with 77 out of 241 (32%) men experiencing three or more barriers to healthcare engagement, compared to 16 out of 172 (9.3%) men over 70 years. Multivariable analysis showed that younger age was associated with higher psychosocial barriers to help-seeking.

**Conclusions:**

Age is a significant factor in men’s health help-seeking preferences in Australia and these findings highlight the unique help-seeking profile of younger men. Younger Australian men are less likely to seek help proactively, and encounter more practical and psychosocial barriers than older men. The findings underscore the necessity for public health strategies to engage younger men effectively in proactive health management.

## Introduction

The health of Australian males is a critical issue that reflects a wider global challenge, necessitating urgent action. Australian men, on average, experience nearly five years less of ‘healthy life’ than women and face higher rates of mortality due to preventable diseases and suicide. [1–4] A crucial factor in these poorer health outcomes experienced by men is a reduced rate of regular and timely medical help-seeking and healthcare utilisation. [5,6] The Australian Government’s National Men’s Health Strategy (NMHS) 2020–2030 aims to address these disparities by enhancing the health system’s capacity to engage with and care for Australian men. [7] This strategy underscores the urgent need to understand and improve the ways that men use health services, and how these services can best meet the physical and mental health needs of men. [7]

Research from Australia and a number of other countries indicates that men’s health help-seeking behaviours are shaped by complex interactions between individual beliefs, social influences, and systemic and societal factors. [8–10] For instance, social support plays a nuanced role in men’s engagement with health services. In some contexts, men may fear that seeking help might lead to a loss of status or ostracism from their peers, particularly in environments where vulnerability is stigmatised. [8] However, in communities where male help-seeking is positively valued, social support can encourage men to access services. [8, 11] The attitudes held by a man’s social network can therefore either facilitate or hinder help-seeking behaviours, highlighting the importance of contextual factors in shaping outcomes. Other potential barriers, such as limited health literacy, and poor service accessibility (e.g., due to cost, location, or privacy concerns) add further complexity to men’s help-seeking. [8–10]

While existing research has provided valuable insights into these factors, much of it relies on qualitative methods with small sample sizes or a focus on specific populations and health conditions. [8–10] This limits the ability to generalise across diverse populations or examine the broader influence of socio-demographic factors on men’s help-seeking behaviours. Preliminary population-level research highlights the important role of socio-demographic factors, particularly age, as moderators of men’s attitudes and behaviours toward healthcare. [6, 12–14] Younger men, for instance, are less likely to hold positive attitudes toward preventive healthcare practices or attend regular health check-ups, potentially contributing to lifelong disengagement from health services. [6,12,15] Furthermore, age is a factor related to how masculine attitudes interact with help-seeking behaviours. [14] While masculine traits like self-reliance and independence have been viewed as inconsistent with the vulnerability required to seek professional help, [16] evidence suggests that these same traits can positively influence healthcare engagement in certain contexts. [14] For example, older men may associate maintaining their health with their ability to provide for their families, leading them to actively seek medical support when needed. [17,18] These findings underscore the importance of exploring how socio-demographic factors, including age, shape men’s perceptions of and approaches to healthcare.

Despite these insights, there remains a gap in the literature regarding how various socio-demographic factors are related to men’s approaches to health help-seeking and the barriers they face, at the population level. This study aims to address this gap by examining: i.) How are age and other socio-demographic characteristics related to help-seeking preferences for physical and mental health in Australian men?; and, ii.) What are the key psychosocial and practical barriers to healthcare use, and how do these vary across different age groups and socio-demographic factors? This investigation can provide an evidence base to guide strategies to promote increased engagement with health services by men across the life course, ultimately contributing to the reduction of health disparities and improvement of men’s health outcomes.

## Methods

### Study design

This study used a national cross-sectional survey of Australian men, with ethics approval obtained from the Monash University Human Research Ethics Committee (Approval No. 27289). The study adheres to Strengthening the Reporting of Observational Studies in Epidemiology (STROBE) guidelines. [19]

### Participants and sampling

Study participants were English-speaking males aged 18 years and older with access to both telephone and the internet. Men were recruited through the Life in Australia (LiA) panel, [20] established by the Social Research Centre in 2016. The LiA panel comprises a probabilistic sample of approximately 4,000 Australian adults enlisted using a dual-frame random digit dialling (DFRDD) method with a 30:70 split between landline and mobile phone numbers. [21]

To ensure representativeness, enrolment weights were calculated using design and post-stratification processes, aligning the LiA sample with Australian population benchmarks. [21] The generalised regression method was used to create the weights, employing non-linear optimisation to minimise weight variation while meeting population totals. [22,23] Data were weighted to the Australian Bureau of Statistics Census profile (2020) [22] across key demographic variables, including age, country of birth, geographic distribution, educational attainment, Socioeconomic Index for Areas (SEIFA) score (a postcode based socioeconomic classification), telephone access, and duration at current residence to reflect the demographic profile of Australians. [24] These survey weighting and DFRDD methods help reduce any biases likely to be introduced through non-coverage and non-response, and improve the generalisability of survey results to the broader Australian male population. [21,22]

All men in the LiA panel (*N* = 1409) were invited through email and text message to complete the survey, with telephone follow-up of non-responders. The invitations included a participant information statement, and written consent was obtained before men continued to complete the survey. Participants received a $10 gift-card for participating in the survey.

### Survey measures

The survey was developed by Healthy Male, a national men’s health agency funded by the Australian Department of Health (see S1 Appendix). The selection of survey measures was informed by consultation with medical and allied health advisors, and a review of Australian and international men’s health surveys. The survey was completed online from 15–29 March 2021.

Participants provided their age, residential postcode (used to classify SEIFA), [25] marital status, educational background, occupation, and country of birth. Participants were also asked about any disabilities and chronic physical or mental health conditions they have.

Help-seeking preferences regarding physical health concerns were measured using items developed by Vincent et al. [26] On a 7-point scale, participants rated their likelihood of engaging in 10 help-seeking actions when experiencing symptoms of physical ill-health, including making an appointment with a doctor immediately, monitoring symptoms to attempt self-diagnosis, and calling a helpline. Participants also rated, using the same scale, their likelihood of seeking help when experiencing ongoing regular pain. Responses were recoded into binary outcomes such that a score of ≥6 (highly likely) was coded as high and <6 (moderate to not likely) coded as low.

In relation to mental health needs, help-seeking preferences were measured using items from the *Ten to Men* Australian longitudinal study on men’s health. [27] Participants rated, on a 7-point scale, their likelihood of 13 different preferences, including mental health professionals, trusted websites, family and friends, or choosing not to seek help at all when experiencing psychological issues. Responses on this scale were again recoded, with a score of ≥6 coded as high and <6 coded as low.

Practical barriers to healthcare access were also measured using items from the *Ten to Men* study. [27] Here, participants were asked if any of 12 listed reasons (e.g., cost, waiting time) had prevented them from accessing necessary healthcare services, including an option for participants to mention unlisted reasons.

The Barriers to Help-Seeking Scale (BHSS) [28] was used to measure psychosocial barriers to help-seeking. This 31-item scale asks participants to assess the significance of different reasons for not seeking help, on a 7-point scale. The BHSS categorises these reasons into five subscales, all of which demonstrate good internal reliability: need for control and self-reliance (α = 0.93); minimising problems and resignation (α = 0.89); concrete barriers and distrust of caregivers (α = 0.79); privacy concerns (α = 0.83); and emotional control (α = 0.89). Mental health-related stigma was assessed using an 8-item subscale of the Inventory of Attitudes toward Seeking Mental Health Services (IASMHS). [29] Here participants rated, on a 5-point scale, their agreement with various reasons for not seeking professional help for psychological problems. Items within the BHSS sub-scales and IASMHS were summed and mean scale scores were calculated, with higher mean scores indicating higher levels of barriers or stigma.

### Statistical analysis

Prevalence calculations were made for physical and mental health help-seeking preferences and practical barriers to seeking healthcare. Bivariate differences were examined using Chi-square, and independent relationships between variables were assessed using forced entry multivariable logistic and linear regression modelling, adjusting for all social-demographic variables as well as self-reported physical and mental health status. Socio-demographic variables included in the analysis were selected based on prior evidence linking these factors to health help-seeking behaviours. [30–36] Statistical analyses were performed using IBM SPSS V28.0.

## Results

### Sample characteristics

Of the 1409 men invited to participate in the survey, 1282 completed the survey (91.0%). Weighted distributions of the socio-demographic profile of the surveyed population are presented in Table 1.

**Table 1:** Characteristics of survey participants (N = 1,282).

Demographic and health factors	n	Unweighted %	Weighted %
**Age (years)**			
18-29	241	8.9	18.8
30-39	270	12.4	21.1
40-49	207	16.2	16.2
50-59	198	18.4	15.5
60-69	189	22.7	14.7
70+	172	21.4	13.5
**Residential location**			
Urban	874	68.2	66.6
Rural	406	31.7	33.4
**Marital status**			
Never married	193	15.1	22.4
Married/partner	943	73.6	67.4
Divorced/widowed	140	10.9	9.9
**SEIFA quintile**			
Q1 (most disadvantaged)	197	15.4	19.1
Q2	214	16.7	20
Q3	240	18.7	19.8
Q4	275	21.5	20.6
Q5 (least disadvantaged)	352	27.5	19.9
**Education**			
University	613	47.9	33.2
Vocational	366	28.6	38.8
High school	262	20.5	28.1
**Occupation**			
Manager/professional	738	57.6	47.5
Trades/manual	321	25.1	30.5
Sales/service	198	15.5	18.8
Other	24	1.9	3.2
**Country of birth**			
English speaking	1066	83.2	77.3
Non-English speaking	212	16.5	22.7
**Disability**			
Living with a disability	313	24.4	22.3
**Chronic disease**			
Physical condition	674	52.6	45.5
Mental condition	191	14.9	16.7

^a^Variables with totals less than 1,282 are due to missing data. ^b^ Data weighted using Australian Bureau of Statistics, 2020, Population estimates. ^c^ Socio-Economic Indexes for Areas.

### Physical health help-seeking preferences

As shown in Table 2, men in the younger and middle-aged age groups (up to 50 years) consistently demonstrated a lower prevalence of help-seeking intentions than men aged 70 and over, except in the use of online resources. Multivariable analysis showed that, compared to those aged 70 and over, the odds ratios (OR) for seeking help from professional sources were lowest for 18–29-year-old men (OR = 0.29, 95% confidence interval (CI) 0.17–0.49), and also lower in the 30–39 years, 40–49 years, 50–59 years and 60–69 years aged brackets. All age groups below 70 years also showed higher odds of using online sources for help, and this was most pronounced among 30–39-year-olds (OR = 2.98, 95% CI 1.76–5.05).

**Table 2:** Logistic regression analyses of associations between socio-demographic characteristics and physical health help-seeking preferences of men.

Population segment	Professional help for symptoms		Online help for symptoms		Family/friend help for symptoms		Self-manage symptoms		Seek help for ongoing pain	
	n (%)	OR (95% CI)	n (%)	OR (95% CI)	n (%)	OR (95% CI)	n (%)	OR (95% CI)	n (%)	OR (95% CI)
**All men**	434 (34)		527 (41)		568 (44)		472 (37)		810 (63)	
**Age group**										
70+	91 (53)	Ref	36 (21)	Ref	75 (43)	Ref	40 (23)	Ref	132 (77)	Ref
18-29	57 (24)	0.28 (0.17-0.49)**	119 (50)	2.34 (1.34-4.10)*	118 (49)	0.88 (0.53-1.46)	108 (45)	3.06 (1.77-5.28)**	151 (63)	0.39 (0.23-0.68)**
30-39	72 (27)	0.35 (0.22-0.58)**	144 (53)	2.98 (1.76-5.05)**	131 (49)	0.85 (0.53-1.36)	141 (52)	4.13 (2.47-6.90)**	157 (59)	0.38 (0.23-0.63)**
40-49	63 (30)	0.44 (0.27-0.71)**	98 (47)	2.54 (1.50-4.30)**	95 (46)	0.91 (0.57-1.45)	78 (38)	1.98 (1.18-3.30)*	121 (59)	0.38 (0.23-0.63)**
50-59	67 (34)	0.51 (0.33-0.81)*	65 (33)	1.70 (1.01-2.87)*	76 (38)	0.74 (0.47-1.17)	60 (30)	1.48 (0.89-2.45)	119 (60)	0.44 (0.27-0.72)*
60-69	84 (44)	0.80 (0.51-1.24)	65 (35)	2.00 (1.19-3.36)*	73 (39)	0.76 (0.48-1.19)	45 (24)	1.14 (0.68-1.91)	130 (69)	0.61 (0.37-1.00)
**Location**										
Urban	293 (34)	Ref	408 (48)	Ref	396 (47)	Ref	313 (37)	Ref	554 (65)	Ref
Rural	141 (33)	0.79 (0.59-1.06)	121 (28)	0.57 (0.42-0.76)**	171 (40)	0.82 (0.62-1.08)	159 (37)	1.14 (0.85-1.51)	259 (61)	0.71 (0.54-0.94)*
**Marital status**										
Never married	89 (31)	Ref	137 (48)	Ref	132 (46)	Ref	127 (45)	Ref	184 (65)	Ref
Divorced/widowed	54 (43)	0.80 (0.48-1.33)	33 (26)	0.57 (0.33-0.97)*	29 (23)	0.33 (0.19-0.57)**	41 (33)	0.91 (0.54-1.54)	76 (60)	0.72 (0.43-1.19)
Married/defacto	291 (34)	0.68 (0.48-0.96)*	356 (41)	0.84 (0.60-1.17)	406 (47)	0.97 (0.70-1.34)	304 (35)	0.96 (0.69-1.33)	548 (64)	0.78 (0.56-1.10)
**SEIFA quintile**										
Q1 (most disadvantaged)	95 (39)	Ref	91 (37)	Ref	109 (45)	Ref	85 (35)	Ref	150 (62)	Ref
Q2	93 (36)	0.98 (0.67-1.45)	95 (37)	0.95 (0.63-1.42)	106 (41)	0.83 (0.57-1.22)	99 (39)	1.14 (0.77-1.68)	168 (66)	1.24 (0.84-1.81)
Q3	77 (30)	0.74 (0.50-1.11)	108 (42)	1.26 (0.84-1.87)	108 (43)	0.77 (0.53-1.12)	97 (38)	1.04 (0.70-1.54)	158 (63)	1.22 (0.83-1.79)
Q4	93 (35)	0.93 (0.64-1.36)	115 (44)	0.99 (0.67-1.46)	129 (49)	1.01 (0.70-1.46)	109 (41)	1.21 (0.83-1.78)	163 (62)	1.07 (0.73-1.55)
Q5 (least disadvantaged)	75 (29)	0.70 (0.47-1.06)	120 (47)	0.89 (0.59-1.34)	113 (44)	0.72 (0.48-1.05)	81 (32)	0.74 (0.49-1.13)	169 (66)	1.08 (0.72-1.61)
**Education**										
University	126 (31)	Ref	220 (54)	Ref	191 (47)	Ref	151 (37)	Ref	257 (63)	Ref
Trade/vocational	168 (35)	1.00 (0.71-1.41)	160 (33)	0.75 (0.54-1.03)	197 (41)	1.12 (0.82-1.54)	164 (34)	1.23 (0.88-1.71)	303 (63)	0.96 (0.69-1.33)
High school	123 (35)	1.08 (0.75-1.54)	139 (40)	0.92 (0.66-1.29)	160 (46)	1.25 (0.89-1.74)	142 (41)	1.65 (1.17-2.34)*	230 (66)	1.01 (0.71-1.42)
**Occupation**										
Manager/professional	198 (33)	Ref	306 (50)	Ref	291 (48)	Ref	235 (39)	Ref	390 (65)	Ref
Trades/manual	143 (37)	1.10 (0.80-1.50)	108 (28)	0.53 (0.39-0.73)**	154 (39)	0.76 (0.56-1.03)	132 (34)	0.78 (0.57-1.07)	241 (62)	1.01 (0.74-1.37)
Sales/service	84 (35)	0.99 (0.70-1.41)	98 (41)	0.78 (0.55-1.09)	104 (43)	0.86 (0.62-1.21)	90 (37)	0.80 (0.57-1.14)	154 (64)	0.95 (0.68-1.34)
**Country of birth**										
English speaking	326 (33)	Ref	372 (38)	Ref	418 (42)	Ref	349 (35)	Ref	637 (65)	Ref
Non-English speaking	108 (37)	1.53 (1.12-2.10)*	154 (53)	1.21 (0.89-1.65)	149 (52)	1.23 (0.91-1.66)	123 (42)	1.11 (0.81-1.52)	173 (60)	0.83 (0.61-1.13)
**Disability**										
No disability	326 (33)	Ref	450 (45)	Ref	461 (46)	Ref	379 (38)	Ref	624 (63)	Ref
Lives with disability	108 (38)	0.97 (0.71-1.33)	80 (28)	0.64 (0.46-0.89)	107 (37)	0.81 (0.60-1.11)	93 (33)	1.05 (0.76-1.45)	189 (66)	1.03 (0.75-1.41)

Note. ^a^ Occupation category ‘Other’ not included due to small sample size. All analyses adjusted for all socio-demographic variables and self-reported physical and mental health status. *p < 0.05; **p < 0.001.

Additionally, men across the 18–29, 30–39, 40–49, and 50–59 year age groups demonstrated a lower willingness to seek help for ongoing pain compared to those aged 70 and over. The lowest odds of seeking help were observed in the 18–29 (OR = 0.39, 95% CI 0.23 − 0.68) and 30–39 (OR = 0.38, 95% CI 0.23 − 0.63) year age groups. Men in the 18–29, 30–39, and 40–49 year age groups were more likely than the oldest age group to prefer to self-manage symptoms, with the 30–39 year age group exhibiting the highest odds of this behaviour (OR = 4.13, 95% CI 2.47–6.90). Notably, several of these age-related odds ratios exceed established thresholds for practical significance, [37] meaning that these differences are not only statistically significant but also large enough to have meaningful real-world implications.

Other significant geographical and demographic associations with help-seeking preferences are shown in Table 2. Men in rural areas were less inclined to seek help online or for ongoing regular pain compared to their urban counterparts. Married men were less likely to seek professional help than men who had never married, while divorced or widowed men showed a lower tendency to use online resources or consult friends than those never married. Educational background and occupation emerged as significant factors; men with a high school level education were more inclined to self-manage symptoms, whereas those in trade or manual labour occupations showed a lesser likelihood to consult online sources compared with professionals. Men born in non-English speaking countries were more likely to seek professional help than those born in English speaking countries.

### Mental health help-seeking preferences

As shown in Table 3, men in all age groups below 70-years reported a lower preference than the older group for seeking professional help when experiencing psychological difficulties. This was most pronounced in young men aged 18–29 years (OR = 0.28, 95% CI 0.16–0.46). Men in the youngest age groups were more likely to seek help from family or friends compared to men in the oldest age category (18–29: OR = 2.24, 95% CI 1.33–3.78; 30–39: OR = 1.94, 95% CI 1.20–3.13). Additionally, men in the 18–29, 30–39 and 40–49 age groups were more likely than men aged 70 and over to seek help from online sources, with the highest likelihood observed in the 30–39 year age group (OR = 3.48, 95% CI 1.77–6.83). As seen in physical health preferences, many of these age-related odds ratios exceed thresholds for practical significance. [37]

**Table 3:** Logistic regression analyses of associations between socio-demographic characteristics and mental health help-seeking preferences of men.

Population segment	Professional		Online		Family and friends		Helpline		Would not seek help	
	n (%)	OR (95% CI)	n (%)	OR (95% CI)	n (%)	OR (95% CI)	n (%)	OR (95% CI)	n (%)	OR (95% CI)
**All men**	582 (46)		304 (24)		492 (38)		119 (9)		157 (12)	
**Age group**										
70+	105 (61)	Ref	15 (9)	Ref	45 (26)	Ref	15 (9)	Ref	20 (12)	Ref
18-29	90 (37)	0.28 (0.16-0.46)**	80 (33)	2.77 (1.36-5.63)*	131 (54)	2.24 (1.33-3.78)*	19 (8)	0.88 (0.34-2.24)	25 (10)	0.76 (0.35-1.65)
30-39	105 (39)	0.31 (0.19-0.49)**	96 (36)	3.48 (1.77-6.83)**	132 (49)	1.94 (1.20-3.13)*	26 (10)	0.88 (0.36-2.14)	40 (15)	1.51 (0.76-2.98)
40-49	83 (40)	0.30 (0.18-0.48)**	47 (23)	2.09 (1.04-4.18)*	76 (37)	1.57 (0.98-2.51)	25 (12)	1.72 (0.73-4.04)	28 (14)	1.21 (0.60-2.43)
50-59	103 (52)	0.61 (0.39-0.96)**	35 (18)	1.92 (0.96-3.85)	58 (29)	1.26 (0.81-1.99)	19 (10)	1.57 (0.68-3.64)	28 (14)	1.27 (0.66-2.47)
60-69	96 (51)	0.63 (0.40-0.99)*	31 (17)	1.88 (0.94-3.77)	50 (27)	1.04 (0.67-1.62)	15 (8)	1.17 (0.49-2.79)	16 (9)	0.75 (0.37-1.54)
**Location**										
Urban	401 (47)	Ref	242 (28)	Ref	357 (42)	Ref	89 (10)	Ref	96 (11)	Ref
Rural	182 (43)	0.65 (0.49-0.86)*	61 (14)	0.59 (0.42-0.84)*	135 (32)	1.07 (0.81-1.41)	30 (7)	1.11 (0.66-1.84)	61 (14)	1.23 (0.83-1.84)
**Marital status**										
Never married	114 (40)	Ref	86 (30)	Ref	152 (53)	Ref	31 (11)	Ref	45 (16)	Ref
Divorced/widowed	59 (47)	0.78 (0.47-1.29)	18 (14)	0.68 (0.35-1.31)	37 (29)	0.73 (0.44-1.20)	8 (6)	0.41 (0.16-1.08)	8 (6)	0.34 (0.15-0.80)*
Married/defacto	410 (48)	1.07 (0.77-1.49)	198 (23)	0.90 (0.63-1.30)	303 (35)	1.39 (0.99-1.95)	79 (9)	0.65 (0.39-1.10)	104 (12)	0.72 (0.46-1.14)
**SEIFA quintile**										
Q1 (most disadvantaged)	107 (44)	Ref	64 (26)	Ref	91 (37)	Ref	19 (8)	Ref	32 (13)	
Q2	122 (48)	1.26 (0.86-1.84)	56 (22)	0.89 (0.57-1.41)	102 (40)	1.11 (0.76-1.62)	17 (7)	1.08 (0.51-2.26)	35 (14)	0.94 (0.55-1.60)
Q3	116 (46)	1.09 (0.74-1.60)	56 (22)	0.89 (0.57-1.40)	91 (36)	0.86 (0.59-1.26)	23 (9)	1.73 (0.87-3.46)	31 (12)	0.94 (0.55-1.62)
Q4	115 (43)	0.97 (0.67-1.41)	55 (21)	0.59 (0.38-0.93)*	103 (39)	1.21 (0.83-1.78)	25 (9)	1.48 (0.75-2.92)	25 (9)	0.64 (0.36-1.14)
Q5 (least disadvantaged)	118 (46)	1.02 (0.69-1.51)	72 (28)	0.79 (0.51-1.24)	101 (40)	1.27 (0.85-1.89)	35 (14)	2.65 (1.35-5.21)*	32 (13)	0.92 (0.52-1.63)
**Education**										
University	180 (44)	Ref	136 (33)	Ref	189 (46)	Ref	49 (12)	Ref	44 (11)	
Trade/vocational	218 (45)	1.01 (0.73-1.39)	83 (17)	0.76 (0.52-1.10)	163 (34)	0.71 (0.51-0.98)*	33 (7)	0.55 (0.32-0.95)*	60 (13)	1.03 (0.63-1.67)
High school	164 (47)	1.13 (0.81-1.58)	82 (24)	0.91 (0.62-1.33)	127 (37)	0.67 (0.48-0.95)*	31 (9)	0.80 (0.46-1.39)	52 (15)	1.30 (0.79-2.14)
**Occupation**										
Manager/professional	289 (48)	Ref	174 (29)	Ref	250 (41)	Ref	53 (9)	Ref	64 (11)	Ref
Trades/manual	174 (45)	0.79 (0.58-1.07)	61 (16)	0.70 (0.48-1.01)	133 (34)	1.15 (0.84-1.56)	30 (8)	1.61 (0.93-2.75)	49 (13)	1.09 (0.69-1.72)
Sales/service	106 (44)	0.79 (0.57-1.11)	53 (22)	0.83 (0.56-1.23)	89 (37)	1.04 (0.74-1.46)	35 (15)	2.87 (1.69-4.87)**	40 (17)	1.50 (0.94-2.40)
**Country of birth**										
English speaking	444 (45)	Ref	192 (19)	Ref	356 (36)	Ref	72 (7)	Ref	128 (13)	
Non-English speaking	139 (48)	1.40 (1.04-1.90)*	112 (39)	1.73 (1.24-2.40)*	135 (47)	0.90 (0.66-1.23)	46 (16)	2.99 (1.87-4.79)**	28 (10)	0.80 (0.49-1.29)
**Disability**										
No disability	445 (45)	Ref	260 (26)	Ref	399 (40)	Ref	92 (9)	Ref	120 (12)	
Lives with disability	139 (49)	0.91 (0.67-1.23)	44 (15)	0.77 (0.51-1.15)	93 (33)	1.19 (0.87-1.62)	27 (9)	1.34 (0.78-2.30)	37 (13)	1.03 (0.66-1.62)

Note. ^a^ Occupation category ‘Other’ not included due to small sample size. All analyses adjusted for all socio-demographic variables and self-reported physical and mental health status. *p < 0.05; **p < 0.001.

Men in rural areas were less likely to seek professional help than those in urban settings. Divorced men showed a higher likelihood of seeking help than those who had never married. Additionally, men in the second least disadvantaged SEIFA quintile (Q4) were less inclined to use online sources for help compared to men in the most disadvantaged quintile (Q1). Men with a trade or vocational education were least likely to use helplines, and along with those with a high school level education, were also least likely to seek support from family or friends. Conversely, men born in non-English speaking countries reported a preference to consult professional services, use online sources, and contact helplines compared to those from English speaking countries.

### Psychosocial barriers to help-seeking across age groups

Multivariable analysis (Table 4) shows that age was inversely related to most of the psychosocial barriers to seeking help for physical or mental health problems, with younger men reporting higher barriers than older age groups. Specifically, men in the 18–29, 30–39, and 40–49 year age cohorts, reported higher levels of concern about self-reliance and control, indicating a belief that seeking help would compromise their independence. This was most prominent in the 30–39 year age group (β = 0.72, 95% CI 0.43–1.00, p < 0.001).

**Table 4:** Linear regression analyses on association between age and psychosocial barriers to seeking help and mental health stigma of men.

	Need for control and self-reliance		Minimising problems and resignation		Concrete barriers and distrust of caregivers		Privacy		Emotional control		Mental health stigma	
	mean (SD)	β (95% CI)	mean (SD)	β (95% CI)	mean (SD)	β (95% CI)	mean (SD)	β (95% CI)	mean (SD)	β (95% CI)	mean (SD)	β (95% CI)
18-29 years	2.54 (1.44)	0.54 (0.23-0.85)**	3.80 (1.44)	1.00 (0.65-1.35)**	2.75 (1.37)	1.09 (0.81-1.36)**	2.77 (1.44)	0.88 (0.56-1.19)**	3.09 (1.60)	0.81 (0.46-1.17)**	2.39 (0.81)	0.25 (0.06-0.43)*
30-39 years	2.76 (1.32)	0.72 (0.43-1.00)**	4.07 (1.39)	1.24 (0.92-1.57)**	2.73 (1.31)	1.00 (0.74-1.25)**	3.09 (1.40)	1.11 (0.82-1.40)**	3.41 (1.47)	1.05 (0.72-1.38)**	2.46 (0.75)	0.32 (0.15-0.49)**
40-49 years	2.69 (1.47)	0.66 (0.38-0.95)**	3.76 (1.43)	0.96 (0.63-1.29)**	2.54 (1.21)	0.82 (0.56-1.08)**	2.74 (1.47)	0.75 (0.45-1.05)**	3.19 (1.62)	0.85 (0.52-1.18)**	2.54 (0.84)	0.42 (0.24-0.59)**
50-59 years	2.41 (1.40)	0.38 (0.11-0.65)*	3.32 (1.61)	0.52 (0.20-0.83)*	2.34 (1.21)	0.63 (0.38-0.88)**	2.39 (1.42)	0.43 (0.14-0.71)*	2.83 (1.59)	0.52 (0.20-0.84)*	2.37 (0.80)	0.23 (0.06-0.39)**
60-69 years	2.24 (1.29)	0.24 (-0.03-0.52)	3.08 (1.49)	0.28 (-0.03-0.59)	2.01 (1.10)	0.30 (0.06-0.54)*	2.12 (1.27)	0.17 (-0.11-0.45)	2.53 (1.46)	0.23 (-0.08-0.55)	2.29 (0.68)	0.14 (-0.03-0.30)
70 + years	1.98 (1.13)	Ref	2.82 (1.44)	Ref	1.72 (0.93)	Ref	1.91 (1.13)	Ref	2.25 (1.41)	Ref	2.19 (0.74)	Ref

Note. All analyses adjusted for all socio-demographic variables and self-reported physical and mental health status. Higher scores indicate higher levels of barrier/stigma. * p < 0.05; ** p < 0.001.

Across these younger age groups, there was also a greater tendency to minimise health issues, suggesting that they were less likely to regard their problems as serious enough to warrant professional help. This was highest among 30–39-year-olds (β = 1.24, 95% CI 0.92–1.57, p < 0.001). Privacy concerns, reflecting fears of vulnerability or exposure, were similarly elevated in these groups, with the highest levels in the 30–39-year-olds (β = 1.11, 95% CI 0.82–1.40, p < 0.001).

The desire to maintain emotional control, which indicates a reluctance to express emotional distress or seek assistance, was notably higher among these younger men, particularly the 30–39-year-olds (β = 1.05, 95% CI 0.72–1.38, p < 0.001). These age groups reported higher concrete barriers such as financial and logistic constraints, especially 18–29-year-olds (β = 1.09, 95% CI 0.81–1.36, p < 0.001). Stigma associated with mental health help-seeking was a notable factor with the 18–29, 30–39, 40–49, and 50–59 year age groups reporting this experience more often than men aged 70 and over. This indicates that these men may perceive a higher level of judgment and/or shame associated with seeking help for psychological problems.

### Practical barriers to health care across age groups

**Fig 1:**
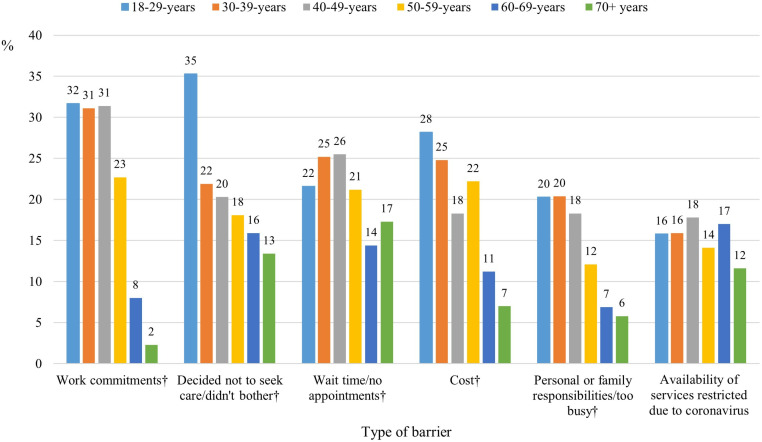
Prevalence of practical barriers to health care in the past 12 months, by age group; Note: † Chi-square, p < 0.05.

Fig 1 presents a breakdown of the most reported practical barriers to healthcare (by at least 10% of respondents) that men in different age groups had experienced in the preceding 12 months. Younger age groups generally indicated more barriers than their older counterparts, with the exception of the COVID-19 impact on service availability (OR presented in S2 Table). Notably, 77 (32%) men aged 18–29 years (OR = 7.56, 95% CI 3.74–15.29) and 86 (32%) men aged 30–39 years (OR = 7.00, 95% CI 3.57–13.71) reported experiencing three or more barriers to healthcare engagement, compared to 16 (9%) men aged 70 and over.

Among younger men, 76 (32%) aged 18–29 years (OR = 33.44, 95% CI 10.36–107.91) and 84 (31%) of those aged 30–39 years (OR = 30.74, 95% CI 9.71–97.34) identified work commitments as an impediment; in contrast, only 4 men (2%) aged 70 and over identified this as a barrier. Similarly, deciding not to seek care was a barrier predominantly reported by the youngest group, with 85 men (35%) aged 18–29 identifying this, whereas less than 22% of men 30 years and older considered it a concern. Cost was a barrier reported by 68 men (28%) aged 18–29 years (OR = 13.29, 95% CI 5.82–30.38) and 67 men (25%) aged 30–39 years (OR = 8.79, 95% CI 3.95–19.53), in contrast with only 21 (7%) of those aged 70 and over. Lastly, personal or family responsibilities were reported as barriers more frequently by young and middle-aged men, with 49 (20%) of those aged 18–29, 55 (20%) of those aged 30–39 and 38 (18%) of the 40–49 age bracket indicating this challenge. While the impact of COVID-19 on service availability was a barrier reported by more than 10% of participants, there were no age-related differences. These age-related odds ratios also exceeded the thresholds for practical significance. [37]

## Discussion

This study underscores the significance of age as a factor in men’s health help-seeking preferences and barriers in Australia. Our findings reveal a notable reluctance among younger men to seek professional help for both physical and mental health problems. Importantly, while older men may have a higher burden of chronic health conditions, [38] our analysis adjusted for these and other socio-demographic factors. Therefore, the observed reluctance among younger men to engage with healthcare services remains a key concern. These findings align with existing research that identifies young men as a demographic group with lower rates of professional help-seeking, particularly for mental health difficulties, and a tendency towards behaviours that delay early disease prevention. [8,12,15,26,39] The consistency and magnitude of the observed age-related differences, with many odds ratios exceeding established thresholds for meaningful effect sizes, [37] reinforce the real-world implications of these results. This underscores the importance of policymakers and healthcare providers to engage closely with young men to better understand their needs and enhance services in ways that promote stronger healthcare engagement. Strengthening these connections is crucial for designing policies and services that are both accessible and aligned with young men’s preferences and experiences.

The preference among younger men for seeking help from online sources and personal networks presents an opportunity to tailor health service engagement strategies. Potential interventions could include peer support services led by men who have experienced similar health challenges. These initiatives have been found to not only increase health literacy and treatment adherence but have also demonstrated effectiveness for men dealing with emotional difficulties and cancer. [40,41] Such programs have been delivered through various channels, including sports clubs, religious organisations, and community events [42,43] offering lower barriers to entry for younger individuals. [44] Additionally, leveraging e-Health as an initial point of contact for professional care may facilitate healthcare engagement. Text messaging and educational websites have shown promise in promoting help-seeking behaviours in relation to sexual and mental health among men. [45,46] Furthermore, engaging a personal support person, such as a family member or close friend, may encourage attendance at healthcare appointments, and adherence to treatments. [47,48]

Psychosocial barriers, including stigma and concerns over emotional vulnerability, present additional challenges to promoting timely help-seeking by young men. These findings suggest that targeted interventions, particularly those that promote health services in ways that align with masculine attitudes, could be instrumental in encouraging healthcare engagement. For example, using campaigns that reframe help-seeking as a strength rather than a weakness could be instrumental in promoting more proactive health behaviours. [17,49] Such campaigns might leverage situations where masculine values could motivate rather than deter health service engagement, such as when men see their health as tied to their productivity and ability to contribute to family life. [17,18]

The identification of practical barriers, particularly among younger men, such as work commitments, cost, and personal responsibilities, is congruent with previous research [8] and emphasises the necessity for healthcare systems to adapt to the needs and lifestyles of this group. These findings reinforce the need for policymakers and healthcare services to remain attuned to the structural challenges young men face, recognising that awareness campaigns and information distribution are unlikely to be sufficient to alter patterns of help seeking and healthcare engagement. Solutions such as flexible scheduling, telehealth services, and workplace or community-based health initiatives could mitigate these barriers, making health services more accessible and appealing to younger men. [45,50,51] Further research is warranted to determine which flexible arrangements are most effective in alleviating these structural barriers and ensuring that interventions are tailored to the specific needs and preferences of younger men.

While our study shows consistent patterns across age groups, it also sheds light on the complexities of men’s health help-seeking, revealing that a variety of socio-demographic characteristics are related to their preferences. Factors like country of birth, education status, and marital status were all independently associated with selected help-seeking preferences. Although this study’s scope did not allow for a deeper investigation, the intersecting effects, and mechanisms through which these factors may influence help-seeking warrant further investigation.

This study’s strength lies in its use of probabilistic sampling and population weighting, ensuring a sample representative of Australian men. However, limitations include the exclusion of non-English speakers, populations without internet or phone access and low representation of men from rural locations. As a result, these possible geographical and cultural moderators of help-seeking behaviours were not accounted for. Additionally, potential biases may be introduced by the reliance on self-reported data, which may be subject to social desirability biases. [52] Nevertheless, the risk of such biases is reduced by the anonymous data collection process. [53]

This study provides novel insights into the distinct help-seeking preferences among men across various age groups. The urgency of addressing these factors is compounded by men’s use of health services, which decreases during adolescence, undermining early diagnosis and intervention to prevent morbidity. [10,15,54,55] By offering a population-level analysis that delineates the specific help-seeking preferences and barriers of men at different life stages, this paper makes a significant contribution to the body of research informing the NMHS. This clearly highlights that the development of targeted help-seeking interventions for young men must be a priority. This is crucial, as effectively engaging young men can set a foundation for health behaviours that persist throughout life, potentially redressing the burden of preventable disease, injury and mortality among men that has consistently been shown in population health surveillance.

## Supporting information

S1 Appendix National Men’s Health Survey Questionnaire. (DOCX)

S2 Table Logistic regression analyses on association between socio-demographic characteristics and practical barriers to healthcare engagement for men. Note. ^a^ Occupation category ‘Other’ not included due to small sample size. All analyses adjusted for all socio-demographic variables and self-reported physical and mental health status. *p < 0.05; **p < 0.001.
(DOCX)
