# Supplementary material for: Age-related differences in men’s preferences and barriers to healthcare: Insights from a national Australian survey
Source: PLoS One. 2025 May 23;20(5):e0323733. doi: 10.1371/journal.pone.0323733 (PMC12101861; doi:10.1371/journal.pone.0323733)
Supplement: S1 Appendix — (DOCX) [file pone.0323733.s001.docx]

2650 National men’s health survey – Questionnaire – March 2020

PROJECT NOTES

- Data collection in Life in Australia™ (Project number = 2650)
- Online only (i.e., no CATI)
- Males only

GENERAL PROGRAMMING NOTES

- Display all grid questions as a grid (i.e., table). Where grids have more than 6 statements, split on separate screens, with 6 statements per screen. Ensure question text and response scale is repeated on each screen.
- For INTERVIEWMODE=WEB, unless specified, hide options 98 and 99. If respondent does not answer, please then add them to the frame
  - MESSAGE ON POP-UP: You have not provided a response. Is that because you’re not sure, or you would prefer not to answer?

***CALL OUTCOMES AND RR1**

**USE STANDARD CALL OUTCOMES

**USE STANDARD RR1

*(INTERVIEWMODE=WEB AND p_gender=males).

MODULE A: Healthy Males questions

*(ALL)

HM Welcome to the National Men’s Health section of the survey. This section is on behalf of Healthy Male and aims to understand your attitudes towards men’s health.

The ethical aspects of this research have been approved by the Monash Research Ethics Committee (Project ID 27289).

To gain information about this survey and decide whether to take part, please read the Explanatory Statement before starting the survey: [INSERT LINK TO EXPLANATORY STATEMENT].

Please click ‘Next’ to complete the survey.

MODULE A: CURRENT HEALTH

*(ALL)

HM_A1. To start with, we would like to ask about your current health.

In general, how would you describe your **physical** health?

1. Poor

2. Fair

3. Very good

4. Excellent

98. Not sure [SHOW ON SCREEN]

99. Prefer not to say [SHOW ON SCREEN]

*(ALL)

HM_A2. In general, how would you describe your **mental** health?

1. Poor

2. Fair

3. Very good

4. Excellent

98. Not sure [SHOW ON SCREEN]

99. Prefer not to say [SHOW ON SCREEN]

*(TS1)

MODULE B: HEALTH AND WELL-BEING ISSUES

*(ALL)

HM_B1. We would now like to ask about health and well-being issues that might concern you.

Below is a list of possible health concerns for men. Which of the three are most concerning to you?

*Please select three responses.*

[MULITIPLE] [MUST SELECT 3] [RANDOMISE 1-19]

1. Smoking

2. Alcohol intake

3. Depression

4. Anxiety

5. Overweight and obesity

6. Lack of exercise

7. Poor diet

8. Erectile difficulty

9. Premature ejaculation

9. Loneliness

10. Stress

11. Strains in personal relationships

12. Challenges in parenting children

13. Body image

14. Fertility

15. Sexually transmitted infections

16. Recreational drug use

17. Performance-enhancing drug use

18. Health and safety at work

19. Managing anger

96. Other issue (Please specify)

98. Not sure

99. Prefer not to say

*(HM_B1=1-96, HAVE CONCERNS)

HM_B2. How satisfied or dissatisfied are you with the information available on the following health issues?

Please rate your level of satisfaction using a 1 to 7 scale, where 1=not at all satisfied, 4=moderately satisfied, 7=completely satisfied.

[STATEMENTS] [ONLY SHOW CODES SELECTED AT B1]

a. Smoking

b. Alcohol intake

c. Depression

d. Anxiety

e. Overweight and obesity

f. Lack of exercise

g. Poor diet

h. Erectile difficulties

i. Premature ejaculation

j. Loneliness

k. Stress

l. Strains in personal relationships

m. Challenges in parenting children

n. Body image

o. Fertility

p. Sexually transmitted infections

q. Using recreational drugs

r. Using performance-enhancing drugs

s. Health and safety at work

t. Managing anger

u. <PIPE IN ANSWER FROM Q3=96>

[RESPONSE FRAME]

1. 1 – Not at all satisfied

2. 2

3. 3

4. 4 – Moderately satisfied

5. 5

6. 6

7. 7 – Completely satisfied

98. Not sure

99. Prefer not to say

*(ALL)

HM_B3. When you have health concerns or begin to experience symptoms of ill health, how likely are you to do each of the following?

On a scale of 1-7, please indicate how likely you are to do each of these things, where 1=not at all likely; 4=somewhat likely; 7=extremely likely.

[STATEMENTS]

a. Call a telephone helpline

b. Visit a trusted website

c. Search the internet (using Google or another search engine)

d. Talk to a friend/colleague before deciding whether to visit a Doctor

e. Talk to a pharmacist/allied health professional to assess the need to visit a Doctor

f. Wait until symptoms are unbearable or prevent you from functioning before visiting a Doctor

g. Monitor the symptoms and try to self-diagnose

h. Monitor the symptoms in hopes they will go away on their own

i. Make an appointment with the Doctor right away

j. Talk to a partner or family member to assess the need to visit a Doctor

[RESPONSE FRAME]

1. 1 – Not at all likely

2. 2

3. 3

4. 4 – Somewhat likely

5. 5

6. 6

7. 7 – Extremely likely

98. Not sure [SHOW IN GRID]

99. Prefer not to say [SHOW IN GRID]

*(ALL)

HM_B4. Now, imagine that you begin to experience pain that is not so overwhelming that you cannot function. However, it continues for more than a few days and you notice it regularly.

How likely would you be to seek help for this health problem?

Please select a number to indicate your answer, where 1=not at all likely, 4=somewhat likely, 7=extremely likely.

1. 1 – Not at all likely

2. 2

3. 3

4. 4 – Somewhat likely

5. 5

6. 6

7. 7 – Extremely likely

98. Not sure [SHOW ON SCREEN]

99. Prefer not to say [SHOW ON SCREEN]

*(ALL)

HM_B5. Below are some reasons why you might not seek help. Please read each reason and decide how important it is in preventing you from seeking help.

If you think that a reason is very important in preventing you from seeking help, you should select 5. If you think that a reason is not at all important, you should select 1. Or you can select any number in between to indicate how important that reason is for not seeking help.

[STATEMENTS]

a. I would think less of myself for needing help.

b. The problem wouldn’t seem worth getting help for.

c. People typically expect something in return when they provide help.

d. Privacy is important to me, and I don’t want other people to know about my problems.

e. I don’t like to get emotional about things.

f. I don’t like other people telling me what to do.

g. The problem wouldn’t be a big deal; it would go away in time.

h. I would have real difficulty finding transportation to a place where I can get help.

i. This problem is embarrassing.

j. I don’t like to talk about feelings.

k. Nobody knows more about my problems than I do.

l. I wouldn’t want to overreact to a problem that wasn’t serious.

m. I wouldn’t know what sort of help was available.

n. I don’t want some stranger touching me in ways I’m not comfortable with.

o. I’d rather not show people what I’m feeling.

p. I’d feel better about myself knowing I didn’t need help from others.

q. Problems like this are part of life; they’re just something you have to deal with.

r. Financial difficulties would be an obstacle to getting help.

s. I don’t like taking off my clothes in front of other people.

t. I wouldn’t want to look stupid for not knowing how to figure this problem out.

u. I don’t like feeling controlled by other people.

v. I’d prefer just to tolerate it rather than dwell on my problems.

w. I don’t trust doctors and other health professionals.

x. I wouldn’t want someone of the same sex touching my body.

y. It would seem weak to ask for help.

z. I would prefer to wait until I’m sure the health problem is a serious one.

aa. A lack of health insurance would keep me from seeking help.

ab. I like to make my own decisions and not be too influenced by others.

ac. I like to be in charge of everything in my life.

ad. Asking for help is like surrendering authority over my life.

ae. I do not want to appear weaker than my peers.

af. I would prefer not to find out that I have a health problem

[RESPONSE FRAME]

1. 1 – Not at all a reason

2. 2

3. 3

4. 4

5. 5

6. 6

7. 7 – Very important reason

98. Not sure [SHOW IN GRID]

99. Prefer not to say [SHOW IN GRID]

*(ALL)

HM_B6. Below is a list of reasons why some people might find it difficult to access health care when they need it.

During the **past 12 months**, have you been prevented from accessing health care for any of the following reasons?

*Please select all that apply.*

[MULITIPLE] [RANDOMISE 1-15]

1. No service available in my area at the time needed

2. Waiting time too long/no appointments

3. Not taking new patients

4. Cost

5. Decided not to seek care/didn't bother

6. Personal or family responsibilities/too busy

7. Work commitments

8. Transportation problems

9. Difficult to talk with my doctor

10. Doctor does not provide information I understand

11. Cannot find a doctor who speaks my preferred language (if not English)

12. Not enough time in an appointment to discuss my concerns

13. Not able to leave the house due to coronavirus restrictions

14. Availability of services restricted due to the coronavirus

15. Lack of telehealth options for the health care needed

96. Other (Please specify)

98. Not sure [SHOW ON SCREEN]

99. Prefer not to say [SHOW ON SCREEN]

*(ALL)

HM_B7. A comprehensive health check involving blood tests and other assessments would allow your Doctor to determine your health status and risk of disease.

How much (in dollars) do you think would be the maximum you would be willing to pay in out-of-pocket expenses for a comprehensive health check?

1. $ [ENTER NUMERIC RESPONSE, NO DECIMAL PLACES, LIMIT TO 4 DIGITS]

98. Not sure

99. Prefer not to say

*(TS2)

MODULE C: EMOTIONAL OR PSYCHOLOGICAL HEALTH

*(ALL)

HM_C1. Now, we would like to ask about seeking help for emotional or psychological difficulties.

When you experience a personal emotional or psychological problem, how likely would you be to seek help from the following?

Using a 1 to 7 scale, where 1=not at all likely, 4=somewhat likely, 7=extremely likely, please indicate how likely you are to use each of these sources of support or information.

[STATEMENTS]

a. Intimate partner (e.g. girlfriend, boyfriend, husband, wife, de facto)

b. Work colleague

c. Friend

d. Parent

e. Other relative/family member

f. Mental health professional (i.e. psychologist, social worker, counsellor)

g. Lifeline

h. Headspace

i. MensLine Australia

j. Doctor/GP

k. Religious leader (e.g. priest, rabbi, chaplain)

l. A trusted website (e.g. BeyondBlue)

m. Searching the internet (using Google or another search engine)

n. Would not seek help

[RESPONSE FRAME]

1. 1 – Not at all likely

2. 2

3. 3

4. 4 – Somewhat likely

5. 5

6. 6

7. 7 – Extremely likely

98. Not sure [SHOW ON SCREEN]

99. Prefer not to say [SHOW ON SCREEN]

*(ALL)

HM_C2. The next questions concern your views about seeking professional help for psychological problems.

The term ‘professional’ refers to individuals such as psychologists, psychiatrists, social workers, and general practitioners, who have been trained to help people deal with psychological problems such as mental health concerns, emotional problems, mental troubles, and personal difficulties.

For each statement below, please indicate how much you agree or disagree.

[STATEMENTS]

a. I would not want my significant other (spouse, partner, etc.) to know if I were suffering from psychological problems

b. Having a psychological problem carries with it a burden of shame

c. Important people in my life would think less of me if they were to find out that I was experiencing psychological problems

d. I would be uncomfortable seeking professional help for psychological problems because people in my social or business circles might find out about it

e. Having been diagnosed with a psychological problem is a blot on a person's life

f. I would feel uneasy going to a professional because of what some people would think

g. Had I received treatment for psychological problems, I would not feel that it ought to be "covered up"

h. I would be too embarrassed if my neighbour saw me going into the office of a professional who deals with psychological problems

[RESPONSE FRAME]

1. Strongly disagree

2. Disagree

3. Neither agree nor disagree

4. Agree

5. Strongly agree

98. Not sure [SHOW ON SCREEN]

99. Prefer not to say [SHOW ON SCREEN]

*(ALL)

HM_C3. We would now like to ask about the personal support that is available to you.

Please rate each statement below, using the following scale to indicate whether you: 1=very strongly disagree; 2=strongly disagree; 3=disagree; 4=neither agree nor disagree; 5=agree; 6=strongly agree; 7=very strongly agree.

[STATEMENTS]

a. There is a special person who is around when I am in need

b. There is a special person with whom I can share my joys and sorrows

c. My family really tries to help me

d. I get the emotional help and support I need from my family

e. I have a special person who is a real source of comfort to me

f. My friends really try to help me

g. I can count on my friends when things go wrong

h. I can talk about my problems with my family

i. I have friends with whom I can share my joys and sorrows

j. There is a special person in my life who cares about my feelings

k. My family is willing to help me make decisions

l. I can talk about my problems with my friends

[RESPONSE FRAME]

1. Very strongly disagree

2. Strongly disagree

3. Disagree

4. Neither agree nor disagree

5. Agree

6. Strongly agree

7. Very strongly agree

98. Not sure [SHOW ON SCREEN]

99. Prefer not to say [SHOW ON SCREEN]

*(ALL)

HM_C4. The next questions are about how you feel about different aspects of your life. For each one, please indicate how often you feel that way, where 1=hardly ever, 2=some of the time; and 3=often.

[STATEMENTS]

a. How often do you feel that you lack companionship?

b. How often do you feel left out?

c. How often do you feel isolated from others?

[RESPONSE FRAME]

1. Hardly ever

2. Some of the time

3. Often

98. Not sure [SHOW ON SCREEN]

99. Prefer not to say [SHOW ON SCREEN]

*(TS3)

MODULE D: MANAGING HEALTH

*(ALL)

HM_D1. The next questions relate to how you manage physical or mental health issues.

On a scale from 1=very difficult to 4=very easy, how easy would you say it is for you to…

[STATEMENTS]

a. Find information on treatments for illnesses that concern you?

b. Find out where to get professional help when you are ill?

c. Understand what your doctor says to you?

d. Understand your doctor’s or pharmacist’s instruction on how to take a prescribed medicine?

e. Judge when you may need to get a second opinion from another doctor?

f. Use information the doctor gives you to make decisions about your illness?

g. Follow instructions from your doctor or pharmacist?

h. Find information on how to manage mental health problems like stress or depression?

i. Understand health warnings about behaviour such as smoking, low physical activity and drinking too much?

j. Understand why you need health screenings?

k. Judge if the information on health risks in the media is reliable?

l. Decide how you can protect yourself from illness based on information in the media?

m. Find out about activities that are good for your mental well-being?

n. Understand advice on health from family members or friends?

o. Understand information in the media on how to get healthier?

p. Judge which everyday behaviour is related to your health?

[RESPONSE FRAME]

1. Very difficult

2. Difficult

3. Easy

4. Very easy

98. Not sure [SHOW ON SCREEN]

99. Prefer not to say [SHOW ON SCREEN]

*(ALL)

HM_D2. From the list of health information sources below, please select the **three (3)** that are most useful for you.

*Please select three responses.*

[MULITIPLE] [MUST SELECT 3] [RANDOMISE 1-18]

1. Websites of health organisations (e.g. Department of Health)

2. Web browsing (e.g. using Google)

3. Facebook

4. Twitter

5. Reddit

6. Instagram

7. Telephone helplines

8. Face-to-face talks/seminars

9. Fact sheets

10. Brochures/booklets

11. Apps (on mobile phone/tablet device)

12. Videos (e.g., YouTube)

13. Podcasts

14. Magazines

15. Advice from a Doctor

16. Advice from partner

17. Advice from family members

18. Advice from friends/colleagues

96. Other (Please specify)

98. Not sure

99. Prefer not to say

*(HM_D2=1-96, FIND INFORMATION USEFUL)

HM_D3. Please rank the following in order of use to you, where ‘1’ is the **most useful**, ‘2’ is the next most useful, and ‘3’ is the third most useful.

[STATEMENTS] [RANDOMISE] [ONLY SHOW CODES SELECTED AT D2]

1. Websites of health organisations (e.g. Department of Health)

2. Web browsing (e.g. using Google)

3. Facebook

4. Twitter

5. Reddit

6. Instagram

7. Telephone helplines

8. Face-to-face talks/seminars

9. Fact sheets

10. Brochures/booklets

11. Apps (on mobile phone/tablet device)

12. Videos (e.g., YouTube)

13. Podcasts

14. Magazines

15. Advice from a Doctor

16. Advice from partner

17. Advice from family members

18. Advice from friends/colleagues

96. <PIPE IN ANSWER FROM Q3=96>

[RESPONSE FRAME]

1. 1 – Most useful

2. 2

3. 3

98. Not sure

99. Prefer not to say

*(ALL)

HM_D4. The following questions are about how you manage your personal health and well-being.

Thinking about your own health now, please indicate the importance of each of these factors, using a scale from 1=not at all important, 4=somewhat important, to 7= extremely important.

[STATEMENTS]

a. Keeping a healthy body weight

b. Regular physical activity

c. Being in a long-term relationship

d. Visiting your Doctor at least once per year

e. Eating vegetables and fruit every day

f. Discussing prostate checks with my Doctor

g. Getting my blood pressure checked

h. Limiting alcohol intake

i. Having regular skin checks

j. Speaking with a professional if my mental health is bad

k. Screening for colon cancer from age 50 years

l. Having friends to talk about personal things with

[RESPONSE FRAME]

1. 1 – Not at all important

2. 2

3. 3

4. 4 – Somewhat important

5. 5

6. 6

7. 7 – Extremely important

98. Not sure [SHOW ON SCREEN]

99. Prefer not to say [SHOW ON SCREEN]

*(ALL)

HM_D5. To what extent to you think it is possible to prevent the following health issues occurring in your life?

[STATEMENTS]

a. Heart disease

b. High blood pressure

c. Skin cancer

d. Stroke

e. Back pain

f. Road traffic accidents

g. Lung cancer

h. Type 2 diabetes

i. Asthma

j. Erection difficulty

k. Prostate cancer

l. High cholesterol

m. Depression

n. Colon cancer

o. Dementia/memory loss

[RESPONSE FRAME]

1. Not at all preventable

2. Rarely preventable

3. Sometimes preventable

4. Mostly preventable

5. Totally preventable

98. Not sure [SHOW ON SCREEN]

99. Prefer not to say [SHOW ON SCREEN]

*(ALL)

HM_D6. The next statements are about your ability to take care of your health. Please indicate how much you agree or disagree with each.

[STATEMENTS]

a. I manage myself well with respect to my health

b. No matter how hard I try, my health just doesn't turn out the way I would like

c. It is difficult for me to find effective solutions to the health problems that come my way

d. I succeed in the projects I undertake to improve my health

e. I'm generally able to accomplish my goals with respect to my health

f. I find my efforts to change things I don't like about my health are ineffective

g. Typically, my plans for my health don't work out well

h. I am able to do things for my health as well as most other people

[RESPONSE FRAME]

1. Strongly disagree

2. Disagree

3. Neither agree nor disagree

4. Agree

5. Strongly disagree

98. Not sure [SHOW ON SCREEN]

99. Prefer not to say [SHOW ON SCREEN]

*(TS4)

MODULE E: STRESS

*(ALL)

HM_E1. The following questions are about your current wellbeing.

This is a list of different stressful events you might have experienced. For each statement, please indicate how much you agree or disagree.

[STATEMENTS]

a. There have been more problems than positive experiences with my health status in the past 3 months

b. There have been more problems than positive experiences with my finances in the past 3 months

c. There have been more problems than positive experiences with my family/friends in the past 3 months

[RESPONSE FRAME]

1. Strongly disagree

2. Disagree

3. Neither agree nor disagree

4. Agree

5. Strongly disagree

98. Not sure [SHOW ON SCREEN]

99. Prefer not to say [SHOW ON SCREEN]

*(ALL)

HM_E2. The following is list of things that could be contributing to stress in your life.

For each statement, please indicate how much you agree or disagree.

[STATEMENTS]

a. Finding time to relax is difficult for me

b. My needs to work or study keep me from my family or leisure more than I would like

c. My work or school often disrupts other parts of my life (home, health, leisure etc.)

d. Overwork and stress, caused by a need to achieve on the job or in school, affects/hurts my life

[RESPONSE FRAME]

1. Strongly disagree

2. Disagree

3. Neither agree nor disagree

4. Agree

5. Strongly disagree

98. Not sure [SHOW ON SCREEN]

99. Prefer not to say [SHOW ON SCREEN]

*(TS5)

MODULE Z: DEMOGRAPHICS

*(ALL)

HM_Z1. Lastly, we want to know a bit about you to understand the characteristics of our survey respondents.

Could you please select your age group below?

1. 18-24 years

2. 25-29 years

3. 30-34 years

4. 35-39 years

5. 40-44 years

6. 45-49 years

7. 50-54 years

8. 55-59 years

9. 60-64 years

10. 65-69 years

11. 70-74 years

12. 75 years and over

98. Not sure

99. Prefer not to say

*(ALL)

HM_Z2. Are you currently…?

1. Married

2. Living together with a partner

3. Divorced

4. Separated

5. Widowed

6. Never married

98. Not sure

99. Prefer not to say

*(ALL)

HM_Z3. Do you have any children?

In your answer, please consider any biological, step or adopted children you may have.

1. Yes

2. No

98. Not sure

99. Prefer not to say

*(Z3=1, HAVE CHILDREN)

HM_Z4. Please indicate the number of children you have in each of the age groups below:

[STATEMENTS]

a. Children aged under 18 years

b. Children aged 18 years and above

[RESPONSE FRAME] [SHOW AS DROP-DOWN MENU]

1. 1

2. 2

3. 3

4. 4

5. 5 Or more

99. Prefer not to say

*(ALL)

HM_Z5. Please select the option which best describes your current employment status.

1. Employed full-time

2. Employed part-time/casual

3. Looking for work/employment

4. Home duties

5. Student

6. Retired

7. Unable to work due to health problems

96. Other (Please specify)

98. Not sure

99. Prefer not to say

*(ALL)

HM_Z6. What has been your main occupation for most of your life?

1. Manager

2. Professional

3. Technician or trades worker

4. Community or personal service worker

5. Clerical or administrative worker

6. Sales worker

7. Machinery operator or driver

8. Labourer

96. Other (Please specify)

97. Have not worked

98. Not sure

99. Prefer not to say

*(ALL)

HM_Z7. Which language do you mainly speak at home?

If more than one language, indicate the one that is spoken most often.

1. English

2. Mandarin

3. Italian

4. Arabic

5. Cantonese

6. Greek

7. Vietnamese

8. Spanish

9. Hindi

10. Tagalog (Filipino)

11. An Aboriginal language (Please specify)

12. A Torres Strait Islander language (Please specify)

96. Other (Please specify)

98. Not sure

99. Prefer not to say

*(ALL)

HM_Z8. Do you have any of the following physical or mental health conditions, disabilities or illnesses that have lasted or are expected to last 12 months or more?

[STATEMENTS]

a. Autism or autism spectrum condition

b. Breathing problem, such as asthma

c. Blindness or partial sight

d. Cancer

e. Dementia or Alzheimer's disease

f. Deafness or hearing loss

g. Diabetes

h. Heart problem, such as angina

i. Joint problem, such as arthritis

j. Kidney or liver disease

k. Learning disability

l. Mental health condition

m. Neurological condition

n. Stroke related illnesses (which affects your day-to-day life)

[RESPONSE FRAME]

1. Yes

2. No

99. Prefer not to say

*(ALL)

HM_Z9. What is your gender?

1. Male

2. Female

96. Other (Please specify)

98. Not sure

99. Prefer not to say

*(ALL)

HM_Z10. Do you think of yourself as…?

1. Straight

2. Gay

3. Bisexual

96. Other (Please specify)

98. Not sure

99. Prefer not to say

*(TS6)

CLOSING SCRIPT

*(ONLINE)

Thank you for taking the time to participate. This survey was conducted by the Social Research Centre on behalf of Healthy Male.

[DISPLAY IF INCENTIVE=1-3] Your reward will be processed and sent in the next few weeks.

This research study has been carried out in compliance with the Privacy Act and the Australian Privacy Principles, and the information you have provided will only be used for research purposes. Our Privacy Policy is available via our website, [www.srcentre.com.au/research-participants#privacy](http://www.srcentre.com.au/research-participants#privacy)

For further information you can contact the Social Research Centre on 1800 023 040 or [LifeinAus@srcentre.com.au](mailto:LifeinAus@srcentre.com.au).

If you would like to talk to someone about any issues that have arisen from participating in this survey, about how you have been feeling, or if you have any concerns about your mental health, please seek support from one of the services listed below:

beyondblue [www.beyondblue.org.au](http://www.beyondblue.org.au)

Phone: 1300 22 4636

Lifeline [www.lifeline.org.au](http://www.lifeline.org.au)

Phone: 13 11 14

If you have been affected by the ongoing Coronavirus pandemic, please contact one of the agencies above if you have concerns about your mental health, or contact Services Australia for other types of assistance:

Services Australia: <https://www.servicesaustralia.gov.au/individuals/subjects/affected-coronavirus-covid-19>

Your answers have been submitted. You may now close the page.
