# Supplementary material for: Age-related differences in men’s preferences and barriers to healthcare: Insights from a national Australian survey
Source: PLoS One. 2025 May 23;20(5):e0323733. doi: 10.1371/journal.pone.0323733 (PMC12101861; doi:10.1371/journal.pone.0323733)
Supplement: S2 Table — Note. a Occupation category ‘Other’ not included due to small sample size. All analyses adjusted for all socio-demographic variables and self-reported physical and mental health status. *p < 0.05; **p < 0.001. (DOCX) [file pone.0323733.s002.docx]

| Population segment | Waiting time/no appointments | | Cost | | Decided not to seek  care | | Personal   responsibilities/too busy | | Work commitments | | | Restricted services due to COVID | | | 3 or more obstacles | | |  |
| --- | --- | --- | --- | --- | --- | --- | --- | --- | --- | --- | --- | --- | --- | --- | --- | --- | --- | --- |
|  | n  (%) | OR  (95% CI) | n  (%) | OR  (95% CI) | n  (%) | OR  (95% CI) | n  (%) | OR  (95% CI) | | n  (%) | OR  (95% CI) | | n  (%) | OR  (95% CI) | | n  (%) | OR  (95% CI) | |
| **All men** | 271  (21%) |  | 251  (20%) |  | 276  (22%) |  | 188  (15%) |  | | 290  (23%) |  | | 198  (16%) |  | | 311  (24%) |  | |
| **Age group** |  |  |  |  |  |  |  |  | |  |  | |  |  | |  |  | |
| 70+ | 30  (17%) | Ref | 12  (7%) | Ref | 23  (13%) | Ref | 10  (6%) | Ref | | 4  (2%) | Ref | | 20  (12%) | Ref | | 16  (9%) | Ref | |
| 18-29 | 52  (22%) | 1.72  (0.90-3.29) | 68  (28%) | 13.29  (5.82-30.38)** | 85  (35%) | 2.80  (1.49-5.27)** | 49  (20%) | 5.31  (2.29-12.29)** | | 76  (32%) | 33.44  (10.36-107.91)** | | 38  (16%) | 0.92  (0.45-1.91) | | 77  (32%) | 7.56  (3.74-15.29)** | |
| 30-39 | 68  (25%) | 2.17  (1.19-3.93)* | 67  (25%) | 8.79  (3.95-19.53)** | 59  (22%) | 1.48  (0.80-2.75) | 55  (20%) | 4.42  (1.97-9.91)** | | 84  (31%) | 30.74 (9.71-97.34)** | | 43  (16%) | 1.07  (0.54-2.11) | | 86  (32%) | 7.00  (3.57-13.71)** | |
| 40-49 | 53  (26%) | 2.13  (1.18-3.84)* | 38  (18%) | 5.83  (2.60-13.05)** | 42  (20%) | 1.43  (0.78-2.65) | 38  (18%) | 3.94  (1.75-8.83)** | | 65  (31%) | 26.04  (8.25-82.25)** | | 37  (18%) | 1.40  (0.72-2.72) | | 65  (31%) | 5.86  (3.00-11.44)** | |
| 50-59 | 42  (21%) | 1.52  (0.86-2.71) | 44  (22%) | 6.22  (2.85-13.55)** | 36  (18%) | 1.17  (0.63-2.15) | 24  (12%) | 2.21  (0.97-5.05) | | 45  (23%) | 15.77  (5.02-49.55)** | | 28  (14%) | 1.14  (0.58-2.22) | | 47  (24%) | 3.34  (1.73-6.47)** | |
| 60-69 | 27  (14%) | 0.88  (0.48-1.62) | 21  (11%) | 2.56  (1.13-5.80)* | 30  (16%) | 1.08  (0.59-2.00) | 13  (7%) | 1.17  (0.48-2.84) | | 15  (8%) | 4.39  (1.32-14.67)* | | 32  (17%) | 1.54  (0.81-2.91) | | 20  (11%) | 1.25  (0.60-2.57) | |
| **Location** |  |  |  |  |  |  |  |  | |  |  | |  |  | |  |  | |
| Urban | 181  (21%) | Ref | 171  (20%) | Ref | 195  (23%) | Ref | 144  (17%) | Ref | | 212  (25%) | Ref | | 147  (17%) | Ref | | 229  (27%) | Ref | |
| Rural | 88  (21%) | 0.90  (0.64-1.26) | 80  (19%) | 1.28  (0.89-1.83) | 81  (19%) | 0.88  (0.63-1.23) | 44  (10%) | 0.76  (0.50-1.15) | | 78  (18%) | 0.70  (0.50-1.00)* | | 51  (12%) | 0.63  (0.42-0.92)* | | 82  (19%) | 0.71  (0.51-1.00)* | |
| **Marital status** |  |  |  |  |  |  |  |  | |  |  | |  |  | |  |  | |
| Never married | 63  (22%) | Ref | 70  (25%) | Ref | 82  (29%) | Ref | 46  (16%) | Ref | | 75  (26%) | Ref | | 56  (20%) | Ref | | 79  (28%) | Ref | |
| Divorced/widowed | 26  (21%) | 1.17  (0.64-2.14) | 24  (19%) | 1.27  (0.68-2.39) | 26  (21%) | 1.25  (0.70-2.24) | 7  (6%) | 0.41  (0.16-1.06) | | 13  (10%) | 0.67  (0.33-1.37) | | 13  (10%) | 0.34  (0.16-0.72)** | | 22  (18%) | 0.92  (0.49-1.74) | |
| Married/defacto | 179  (21%) | 1.10  (0.74-1.63) | 156  (18%) | 1.06  (0.71-1.56) | 167  (19%) | 0.84  (0.58-1.21) | 134  (16%) | 1.21  (0.79-1.86) | | 201  (23%) | 1.28  (0.89-1.86) | | 128  (15%) | 0.63  (0.42-0.96)* | | 209  (24%) | 1.29  (0.89-1.87) | |
| **SEIFA quintile** |  |  |  |  |  |  |  |  | |  |  | |  |  | |  |  | |
| Q1 (most disadvantaged) | 54  (22%) | Ref | 46  (19%) | Ref | 50  (20%) | Ref | 38  (16%) | Ref | | 59  (24%) | Ref | | 33  (13%) | Ref | | 59  (24%) | Ref | |
| Q2 | 73  (29%) | 1.29  (0.83-1.98) | 47  (18%) | 1.00  (0.60-1.64) | 54  (21%) | 0.96  (0.61-1.52) | 34  (13%) | 0.84  (0.49-1.45) | | 62  (24%) | 0.94  (0.60-1.49) | | 43  (17%) | 1.30  (0.77-2.19) | | 72 (28%) | 1.15  (0.73-1.79) | |
| Q3 | 46  (18%) | 0.64  (0.39-1.03) | 56  (22%) | 1.50  (0.93-2.42) | 57  (22%) | 1.04  (0.66-1.65) | 38  (15%) | 1.02  (0.60-1.73) | | 56  (22%) | 0.67  (0.42-1.07) | | 51  (20%) | 1.81  (1.09-3.00)* | | 65  (26%) | 1.00  (0.64-1.58) | |
| Q4 | 45  (17%) | 0.66  (0.42-1.05) | 48  (18%) | 1.10  (0.68-1.79) | 53  (20%) | 0.85  (0.54-1.34) | 29  (11%) | 0.56  (0.32-0.97)* | | 49  (19%) | 0.51  (0.32-0.81)** | | 32  (12%) | 0.80  (0.46-1.37) | | 55  (21%) | 0.69 (0.44-1.08) | |
| Q5 (least disadvantaged) | 49  (19%) | 0.66 (0.41-1.07) | 54  (21%) | 1.38  (0.84-2.28) | 63  (25%) | 0.98  (0.61-1.56) | 49  (19%) | 0.99  (0.59-1.67) | | 62  (24%) | 0.68  (0.42-1.08) | | 38  (15%) | 0.89  (0.52-1.54) | | 60  (23%) | 0.67  (0.42-1.08) | |
| **Education** |  |  |  |  |  |  |  |  | |  |  | |  |  | |  |  | |
| University | 95  (23%) | Ref | 84  (20%) | Ref | 115  (28%) | Ref | 82  (20%) | Ref | | 121  (29%) | Ref | | 74  (18%) | Ref | | 122  (30%) | Ref | |
| Trade/vocational | 77  (16%) | 0.62  (0.41-0.93)* | 91  (19%) | 1.23  (0.82-1.85) | 96  (20%) | 0.71  (0.49-1.03) | 66  (14%) | 0.88  (0.57-1.36) | | 104  (22%) | 0.90  (0.62-1.32) | | 64  (13%) | 0.70  (0.45-1.08) | | 94  (20%) | 0.72  (0.49-1.05) | |
| High school | 84  (24%) | 1.17  (0.78-1.74) | 66  (19%) | 1.27  (0.84-1.95) | 59  (17%) | 0.46  (0.31-0.70)** | 34  (10%) | 0.63  (0.39-1.03) | | 60  (17%) | 0.72  (0.48-1.09) | | 55  (16%) | 0.90  (0.58-1.40) | | 83  (24%) | 1.07  (0.73-1.57) | |
| **Occupation** |  |  |  |  |  |  |  |  | |  |  | |  |  | |  |  | |
| Manager/professional | 131  (22%) | Ref | 119  (20%) | Ref | 141  (23%) | Ref | 102  (17%) | Ref | | 156  (26%) | Ref | | 97  (16%) | Ref | | 164  (27%) | Ref | |
| Trades/manual | 78  (20%) | 0.97  (0.67-1.42) | 62  (16%) | 0.79  (0.53-1.18) | 68  (17%) | 0.96  (0.66-1.40) | 48  (12%) | 0.97  (0.62-1.49) | | 80  (21%) | 0.84  (0.57-1.22) | | 56  (14%) | 0.96  (0.63-1.46) | | 79  (20%) | 0.74  (0.51-1.06) | |
| Sales/service | 58  (24%) | 1.21 (0.81-1.81) | 65  (27%) | 1.59  (1.06-2.37)* | 53  (22%) | 1.10  (0.73-1.63) | 34  (14%) | 1.07  (0.67-1.71) | | 51  (21%) | 0.81  (0.54-1.23) | | 41  (17%) | 1.10  (0.70-1.71) | | 61  (25%) | 0.95  (0.64-1.41) | |
| **Country of birth** |  |  |  |  |  |  |  |  | |  |  | |  |  | |  |  | |
| English speaking | 206  (21%) | Ref | 173  (18%) | Ref | 214  (22%) | Ref | 129  (13%) | Ref | | 227  (23%) | Ref | | 145  (15%) | Ref | | 224  (23%) | Ref | |
| Non-English speaking | 65  (22%) | 1.06  (0.73-1.53) | 78  (27%) | 2.21  (1.53-3.19)** | 61  (21%) | 0.72  (0.50-1.04) | 59  (20%) | 1.35  (0.90-2.03) | | 62  (21%) | 0.62  (0.42-0.90)* | | 53  (18%) | 1.38  (0.93-2.04) | | 87  (30%) | 1.27  (0.90-1.81) | |
| **Disability** |  |  |  |  |  |  |  |  | |  |  | |  |  | |  |  | |
| No disability | 210  (21%) | Ref | 178  (18%) | Ref | 224  (23%) | Ref | 153  (15%) | Ref | | 232  (23%) | Ref | | 154  (15%) | Ref | | 238  (24%) | Ref | |
| Lives with disability | 61  (21%) | 1.08  (0.74-1.58) | 73  (26%) | 2.50  (1.71-3.67)** | 51  (18%) | 0.83  (0.56-1.21) | 34  (12%) | 0.97  (0.61-1.53) | | 58  (20%) | 1.30  (0.88-1.93) | | 44  (15%) | 0.98  (0.65-1.48) | | 73  (26%) | 1.43  (0.98-2.07) | |
